# Determinants of school-going adolescent girls’ health and nutritional status in district Malir, Karachi: a baseline cross-sectional study

**DOI:** 10.3389/fnut.2024.1510183

**Published:** 2025-01-07

**Authors:** Saira Zafar, Babar Tasneem Shaikh, Zoha Imtiaz Malik, Abdul Momin Rizwan Ahmad

**Affiliations:** ^1^Department of Public Health, Health Services Academy, Islamabad, Pakistan; ^2^Department of Nutrition and Dietetics, National University of Medical Sciences, Rawalpindi, Pakistan

**Keywords:** adolescent girls, dietary behaviors, nutrition education, undernutrition, underweight

## Abstract

**Introduction:**

Adolescence is a phase of life marked by rapid growth. Adequate nutrition is essential during this developmental stage, leading to significant physical performance, improved cognitive ability, and productivity. Improving adolescent girls’ nutrition is crucial for breaking the intergenerational cycle of malnutrition, but research in Pakistan has largely focused on children under five and pregnant women, often neglecting this vulnerable group.

**Objective:**

This study aimed to determine the contextual and sociodemographic factors contributing toward undernutrition to improve the health and nutritional status of adolescent girls.

**Methods:**

A school based cross sectional study was conducted as the baseline survey for a mixed methods study leading to a pilot cluster randomized controlled trial. *n* = 84 girls (10–14 years) from two public schools were selected by simple random sampling. Data were collected through a structured questionnaire including socio demographic profile, anthropometric measurements, dietary patterns, preferences, safe water and hygiene practices, access to health care facilities, and understanding of malnutrition-related symptoms. The data collected were analyzed through SPSS version 27.0. The chi-square test was used to determine the association between the two BMI categories; underweight and normal weight, and various socio-demographic variables.

**Results:**

Among the total of 84 adolescent girls, overall mean height was 143.9 ± 8.1 cm and mean weight was 34.4 ± 6.9 kg, while mean BMI was 16.4 ± 2.2 kg/m^2^. 86.9% of girls were underweight, whereas 13.1% had a normal BMI. The z-scores for both height-for-age and BMI-for-age were in negative values, i.e., −1.62 ± 0.89 and −1.18 ± 1.05, respectively.

**Conclusion:**

Findings of this study demonstrated that the high burden of underweight among school-going adolescent girls needs targeted approaches like nutrition education interventions to enhance awareness about healthy dietary behaviors, food preferences, and ensuring access to safe, sufficient, and healthy diet.

**Recommendation:**

A multipronged approach is required to improve health and nutritional status of adolescent girls by involving individuals, families, education sector, health and other relevant sectors. Larger scale studies are still required for better understanding of the issue and to guide in designing targeted programs to address specific needs of this particular age group.

## 1 Introduction

Nutrition is fundamental to health and growth at every stage of life. From fetal development to old age, it supports survival, physical and mental development, productivity, and overall well-being. Adolescence marks a decisive juncture in life, characterized by rapid growth, requires proper nutrition to ensure healthy development. The consequences of inadequate nutrition during adolescence can resonate throughout the life course, affecting their physiological systems, growth and development and predisposing individuals to nutrition-related chronic ailments in adulthood (1).

A sustainable diet is imperative during this period to avert under nutrition, poor academic performance, compromised health, behavioral and psychological developmental issues, pregnancy related complications, and reduced productivity. Food insecurity seriously affect children and adolescents (2).

Globally, there are approximately 1.3 billion adolescents aged 10–19, with 90% residing in low and middle-income countries (LMICs), where they constitute a significant proportion, representing over 16% of the world’s population (3). Alarmingly, nearly half of adolescents in several countries are at risk of under nutrition, threatening their growth trajectories during this critical phase. Adolescence witnesses a rapid surge in growth, with individuals gaining up to 20% of their adult height, 50% of peak bone mass and 50% of their adult body weight (4).

In 2019, adolescents aged 15–19 in low- and middle-income countries (LMICs) experienced an estimated 21 million pregnancies annually, with about 50% of these pregnancies being unintended, leading to approximately 12 million births, imposing significant strain on their bodies and jeopardizing both their own growth and the well-being of their offspring (5). Adolescent pregnancy perpetuates the intergenerational cycle of malnutrition, particularly under nutrition, contributing to high maternal and child mortality rates with a continuous cycle of compromised health and poverty, especially in low middle income countries with fragile health systems. Adolescent mothers face greater risks of mortality and morbidity during childbirth compared to older counterparts and are more susceptible to nutritional deficiencies (6).

The health and nutritional status of adolescent girls profoundly affect not only their own well-being but also increase the incidence of poor birth outcomes. Efforts must be directed toward providing comprehensive nutritional support, delaying marriage and pregnancy, and breaking the cycle of under nutrition across generations (7). Beyond nutrition, economic conditions, education, social, and environmental factors play pivotal roles in optimal growth during adolescence and support future well-being (8). Social determinants of health have significant impact during this phase, influencing the nutritional status of future generations. Addressing food insecurity, providing access to healthcare and education with emphasis on dietary counseling and dietary diversity, along with familial and interpersonal networks, strongly impact adolescent health (9).

Investing in adolescents through contextually relevant programs and policies can yield substantial dividends in improving health outcomes, particularly in resource-constrained settings. Multisectoral collaboration incorporating health, education, and nutrition sectors is essential to address the multifaceted challenges faced by adolescents and to foster holistic development among individuals, communities, and nations (10).

In South Asia, adolescents make up about 30% of the population, yet they face significant disparities in health, education, and nutrition. In many low- and middle-income countries, adolescent nutrition programs are often neglected, and discriminatory practices around nutrition are widespread. South Asian countries are facing malnutrition crisis with insufficient progress to improve adolescent health and nutritional status. It is crucial to implement effective interventions to improve adolescent nutritional status (11). According to the current estimates, Pakistan is the fifth most populous country in the world and the adolescents’ population is remarkably increasing. 22.7% of the population are adolescent girls. Almost one in eight adolescent girls (11.8%) is underweight, while 56.6% of adolescent girls are anemic requiring utmost attention, while in Sindh province, 16.6% of adolescent girls are underweight, which is the highest of all provinces of Pakistan. Karachi is the biggest metropolitan city of Sindh, Pakistan. 55.7% girls are anemic and 14.9% are underweight in District Malir of Karachi (12).

Under nutrition among adolescents is one of the challenges currently faced by Pakistan, which is threatening productivity of our human capital. It is imperative to design nutrition-related interventions while considering the country’s specific contextual factors, to effectively reduce the high burden of nutritional deficiencies. Key factors include limited resources, food insecurity, lack of education, inadequate health services, individual knowledge and practices related to nutrition, all shaped by the sociocultural, economic, and political environment (13). The context specific multifaceted interventions need to be implemented at the individual, household, and community levels, as well as within the health sector, education, food systems and other relevant sectors (14).

This study aims to identify the factors contributing to adolescent undernutrition by examining contextual influences including socio-cultural dynamics. It seeks to develop culturally sensitive nutrition-related interventions by engaging adolescents, their families, teachers, and communities, using schools as a platform to test the feasibility, acceptability and implementation of these interventions.

## 2 Materials and methods

### 2.1 Study design, period and area

This cross-sectional study was conducted as a baseline survey before initiating the mixed methods study leading to the pilot cluster (RCT) randomized controlled trial for PhD research after approval from the research and ethics committee of health services academy, Islamabad. The study was conducted in two union councils of Gadap, District Malir, Karachi, Sindh in February 2022. The two areas had similar contextual characteristics, socioeconomic status, cultural diversity etc.

### 2.2 Source population, study population and study unit

The source population was adolescent girls (10–14 years of age). Our study population consisted of the adolescent girls enrolled in public schools of two union councils Dumba Goth and Memon Goth of district Malir, Karachi, Sindh, Pakistan. Study units were government schools.

Unmarried Adolescent girls (10–14 years) enrolled in the schools and residents of the selected union councils were included while unmarried adolescent girls (10–14 years) with history of recent acute disease in last 7–10 days were excluded from the study.

### 2.3 Sample size determination, technique, and sampling procedure

By using RaoSoft online sample size calculator, with estimated study population, i.e., adolescent girls of 14,560 (i.e., 22.7% of total population 64,192 according to Census 2017) in Gadap, District Malir, 5% margin of error (confidence interval), 80% study power and a response distribution of 50%, the sample size was calculated to be 375 for randomized controlled trial.

According to Lewis (15), extant literature suggests that a pilot study sample should be 10% of the sample projected for the larger study.

Thus, sample size for the pilot study was: 10% of 375 = 38.

After adjusting it for a 10% loss to follow up, there were 42 participants in each group and 84 participants in total. A simple random sampling technique was used. Unit of randomization were schools. After that eligible students fulfilling inclusion criteria were recruited for the study.

### 2.4 Study variables

The Main outcome variable was nutritional status of adolescent girls evident by their Mid upper arm circumference (MUAC) and Body Mass Index (BMI). Other independent variables were age, family size, family income, educational status of father, educational status of mother, occupation of father, house ownership, house structure, vehicle ownership, household environment including the source of drinking water, household latrine facilities, hand washing, food frequency, food diversity, food security, media exposure etc.

#### 2.4.1 Operational definitions

##### 2.4.1.1 BMI

It is a measure to represent nutritional status in adolescents. It is calculated as person’s weight in kilograms divided by the square of the person’s height in meters (kg/m^2^).

##### 2.4.1.2 MUAC

It is the anthropometric measure used to assess the nutritional status of the individuals by measuring the circumference of the upper arm at the midpoint between the tip of the shoulder and the elbow bent at 90 degree angle by using a MUAC tape (16).

##### 2.4.1.3 Food security

Food security is defined when all people, at all times, have physical and economic access to sufficient safe and nutritious food that meets their dietary needs and food preferences for an active and healthy life (17).

##### 2.4.1.4 Dietary patterns

These are the quantities, proportions, variety, or combination of different foods, drinks, and nutrients in diets, and the frequency with which they are habitually consumed (18).

##### 2.4.1.5 Nutritional status

Nutritional status refers to the evaluation of an individual’s health in terms of their diet, body weight, and biochemical data (19).

### 2.5 Data collection methods

Data were collected from the schools selected randomly in two union councils of Gadap: Dumba Goth and Memon Goth. The list of schools was obtained from the District Education Office, Malir, Karachi. Lists of students were provided by school authorities, and students were selected by simple random sampling.

The structured questionnaires for baseline survey included socio demographic profile, history, anthropometric measurements, dietary behaviors, preferences, access to health care facilities, safe water and hygiene practices, and understanding of malnutrition-like symptoms. The questionnaire was pretested in similar settings.

For anthropometric measurements, standing height was measured using a stadiometer. For measuring height, participants were asked to stand barefoot and look forward with shoulders, buttocks, and heels touching the vertical surface of the stadiometer. Before measuring height, all the participants were asked to fill the rib cage with air. Weight was measured using electronic scales and participants were weighed with light clothing and without shoes. Mid upper arm circumference was measured by nonelastic measuring tape (MUAC tape) to measure the posterior surface of the upper left arm, with the person standing, weight even on both feet, and facing away from the examiner. The elbow of the left arm bent at a 90-degree angle, left palm facing up. The upper-arm length was then measured. The tape was positioned at the shoulder blade and extended to the elbow. With the tape in place, the examiner placed a mark on the posterior surface as the midpoint of the arm. The measuring tape was positioned at the midpoint and wrapped around the arm, without causing a compression of the skin, and was measured to the nearest 0.1 cm.

### 2.6 Data analysis

The data were managed and analyzed using Statistical Package for Social Sciences (IBM SPSS, version 27.0). Descriptive analysis of continuous variables was reported as mean and standard deviation, while categorical data including the baseline socio-demographics, food preferences, food security, dietary patterns and symptoms of malnutrition were described and presented as frequency and percentages. The inferential statistics were performed to compare the nutritional status with baseline survey data. The nutritional status was determined in terms of BMI (as categorical variable with two classes, i.e., normal and underweight), and MUAC (as a continuous variable). The BMI categories were compared with socio-demographic variables, dietary patterns, food preferences, food security and malnutrition symptoms by applying Chi-square test, whereas mean MUAC was compared among the same set of variables by applying Independent-samples T-test or one-way ANOVA depending on the nature of categorical variables. A *p*-value of less than or equal to 0.05 was considered significant.

## 3 Results

### 3.1 Demographic characteristics

Out of 84 study participants, 38 (45.2%) belonged to the intervention group, while 46 (54.7%) were from the control group. All the study participants were school-going adolescent females, with overall mean age of 12.5 ± 1.0 years. Mean age of menarche was reported to be 11.8 ± 2.1. Overall, the mean school year was 6.12 ± 0.6. The mean family size was 7.6 ± 2.5. On average, there were total 3.0 ± 1.7 adults in the family, average number of children was 4.8 ± 2.2, whereas average number of siblings was 4.3 ± 2.0. Table 1 gives a summary of demographic characteristics in detail.

**Table 1:** Summary of demographic, anthropometric and socioeconomic data.

Variables	Mean ± S.D. (*n* = 84)
Age of respondent	12.5 ± 1.0
Age of menarche	11.8 ± 2.1
School year	
Mean	6.12 ± 0.6
Minimum	5
Maximum	7
Family size	
Mean	7.6 ± 2.5
Minimum	3
Maximum	17
Total adults (≥18 years)	
Total mean	3.0 ± 1.7
Males mean	1.5 ± 1.0
Females mean	1.4 ± 0.8
Total children (<18 years)	
Total mean	4.8 ± 2.2
Males mean	1.9 ± 1.2
Females mean	2.8 ± 1.6
Total siblings	
Total mean	4.3 ± 2.0
Males mean	2.1 ± 1.2
Females mean	2.1 ± 1.4
Mid upper arm circumference (cm)	19.8 ± 2.4
Height (cm)	143.9 ± 8.1
Weight (cm)	34.4 ± 6.9
BMI (kg/cm^2^)	16.4 ± 2.2
BMI categories	
Underweight (<18.5)	73 (86.9%)
Normal (18.5–24.9)	11 (13.1%)
Height for age (z-score)	−1.62 ± 0.89
BMI for age (z score)	−1.18 ± 1.05
Father’s/guardian’s occupation	
Shop keeper	14 (17.1%)
Driver	14 (17.1%)
Guard	2 (2.4%)
Daily wager	9 (11.0%)
Employee	43 (52.4%)
Unemployed	2 (2.4%)
Father’s occupation type	
Self employed	50 (59.5%)
Private employee	23 (27.4%)
Government employee	9 (10.7%)
Semi government	2 (2.4%)
Family monthly income (PKR)	20,781 ± 8,547
Minimum	5,000
Maximum	40,000
Are you given any money for your personal expenses each month?	
Never	1 (1.2%)
Rarely	2 (2.4%)
Sometimes	42 (50.6%)
Monthly	23 (27.7%)
Always	15 (18.1%)
If yes, how much (on average)? (PKR)	29.3 ± 16.8
Minimum	0
Maximum	100
House ownership	
Rented	11 (13.1%)
Owned	73 (86.9%)
Housing structure	
Kacha	9 (10.7%)
Pacca	69 (82.1%)
Katcha Pacca	6 (7.1%)
Water supply	
Piped water	75 (89.3%)
Natural spring	1 (1.2%)
Well water	6 (7.1%)
Common water tank	1 (1.2%)
Canal/river	1 (1.2%)
Where do you go to defecate?	
Household latrine	60 (71.4%)
Other latrine	24 (28.6%)
What type of fuel your household mainly uses for cooking?	
Cylinder gas	8 (9.5%)
Sui gas	74 (88.1%)
Straw/shrubs/grass/wood	61 (72.6%)
Do you have birds/animals in the house?	
Yes	39 (46.4%)
No	45 (53.6%)
Do you use products of your own animals in the house?	
Yes	25 (29.8%)
No	59 (70.2%)
Do you have television at home?	
Yes	64 (76.2%)
No	20 (23.8%)
Do you have any transport vehicles at home?	
No	11 (28.9%)
Yes, Motorcycle	58 (72.5%)
Yes, Car	5 (6.3%)
Yes, Cycle	6 (7.5%)
Father’s education	
Not able to read or write	25 (29.8%)
Less than primary	34 (40.5%)
Primary	19 (22.6%)
Secondary	6 (7.1%)
Mother’s education	
Not able to read or write	49 (58.3%)
Less than primary	27 (32.1%)
Primary	8 (9.5%)

### 3.2 Anthropometric measurements

Anthropometric measurements, including weight, height, mid-upper arm circumference, body-mass-index and z-scores for height-for-age and BMI-for age were measured. The overall mean weight was 34.4 ± 6.9 kg, while mean BMI was 16.4 ± 2.2 kg/m^2^.

For BMI categories, majority of the participants, 73 (86.9%) were underweight (BMI <18.5 kg/m^2^), whereas 11 (13.1%) had normal BMI of 18.5–24.9 kg/m^2^. The z-scores for both height-for-age and BMI-for-age were in negative values, i.e., −1.62 ± 0.89 and −1.18 ± 1.05, respectively. Table 1 gives a summary of anthropometric measurements in detail.

### 3.3 Socioeconomic characteristics

Table 1 gives detailed general socioeconomic analysis. It was found that there were overall 50 (59.5%) participants whose father were self-employed, and the mean monthly income was reported to be 20,781 ± 8,547 Pakistani Rupees. On average the mean pocket money was reported to be 29.3 ± 16.8 Rupees with maximum of 100 Rupees.

There were 11 (13.1%) participants living in rented houses, while 73 (86.9%) lived in their own house. The water supply was piped for 75 (89.3%), while 6 (7.1%) used well water as main water source, others including 1 (1.2%) got water from natural spring, 1 (1.2%) common water tank, 1 (1.2%) canal/river for daily use purposes. For defecation, there were 60 (71.4%) participants who reported to have household latrine, while 24 (28.6%) reported other type of latrine which was not located inside the house. Seventy-four (88.1%) participants reported to only use sui gas in the kitchen, while 61 (72.6%) used wood/straw in addition to sui gas for cooking food, and 8 (17.4%) reported to use cylinder gas alone or in addition to sui gas for cooking food. It was found that 11 (28.9%) participants had no vehicle owned at home, 58 (72.5%) had motorbikes, while 5 (6.3%) and 6 (7.5%) had cars and cycle for transportation.

In terms of father’s educational status, overall, there were 25 (29.8%) participants whose fathers were not able to read or write, while fathers of 34 (40.5%) participants had less than primary education, fathers of 19 (22.6%) had primary education and fathers of 6 (7.1%) had secondary education level.

In terms of mothers’ educational status, overall, there were 49 (58.3%) participants whose mothers were not able to read and write, while mothers of 27 (32.1%) participants had less than primary level education, and mothers of 8 (9.5%) participants had primary education as shown in Table 1.

### 3.4 Dietary patterns

It was reported by 72 (85.7%) participants that they think their appetite is good. Most of the participants, 71 (84.5%) reported eating two proper meals on a daily basis most of the times, while 10 (11.9%) used to have three proper meals, and 3 (3.6%) reported to have single proper meal daily as shown in Table 2.

**Table 2:** Dietary patterns, preferences and food security.

Variables	Overall (*n* = 84)
Dietary patterns	
Do you think that you have a good appetite?	
Yes	72 (85.7%)
No	12 (14.3%)
How many meals do you have daily?	
1 meal	3 (3.6%)
2 meals	71 (84.5%)
3 meals	10 (11.9%)
Do you take snacks between regular meals?	
Never	2 (2.4%)
Rarely	6 (7.1%)
Sometimes	54 (64.3%)
Most of the time	20 (23.8%)
Always	2 (2.4%)
Do you skip meals?	
Never	12 (14.3%)
Rarely	2 (2.4%)
Sometimes	65 (77.4%)
Most of time	5 (6.0%)
Which meal do you normally skip?	
Breakfast	6 (8.1%)
Lunch	29 (39.2%)
Dinner	39 (52.7%)
How frequently do you skip your meals?	
Never	10 (11.9%)
Rarely	5 (6.0%)
Sometimes	51 (60.7%)
Most of time	17 (20.2%)
Always	1 (1.2%)
Do you eat junk/street food	
Never	5 (6.0%)
Rarely	8 (9.5%)
Sometimes	40 (47.6%)
Most of time	31 (36.9%)
Food preferences	
Are there any foods you would like to eat more?	
Yes	83 (98.8%)
No	1 (1.2%)
What foods would you like to eat more?	
Meat (Chicken, Beef, Mutton and fish)	59 (70.2%)
Dairy Products	18 (21.4%)
Cereals (wheat, rice, potatoes, Corns)	61 (73.5%)
Eggs	42 (50%)
Vegetables	53 (63.1%)
Pulses	29 (34.5%)
Fruits	61 (72.6%)
Junk Food	26 (31.0%)
Sweets	40 (47.6%)
Have you ever experienced cravings for any non-food items? e.g., mud, chalk, paper, coal etc.	
Yes	4 (4.8%)
No	80 (95.2%)
If yes, what is the craving for?	
Mud/clay	3 (75.0%)
Chalk	1 (25.0%)
Food security	
Do you have enough food at home to eat each day?	
Never	2 (2.4%)
Rarely	1 (1.2%)
Sometimes	9 (10.7%)
Most of time	38 (45.2%)
Always	34 (40.5%)
Have you ever had any food shortage in the house in the last year?	
Never	55 (65.5%)
Rarely	14 (16.7%)
Sometimes	14 (16.7%)
Most of time	1 (1.2%)
Always	0 (0%)
Do all household members have the same number of meals?	
Yes	82 (97.6%)
No, Females have more	0 (0%)
No, Males have more	2 (2.4%)
Do all Household members eat together at mealtimes?	
Yes	77 (91.7%)
No, Females eat first	1 (1.2%)
No, Males eat first	6 (7.1%)
Do all the household members have same quality of food?	
Yes	80 (95.2%)
No, Females have more	0 (0%)
No, Males have more	4 (4.8%)

### 3.5 Food preferences

As a part of the survey, the participants were asked if there is any food that they would like to eat more, in reply to this there were 83 (98.8%) participants who said yes. In respect of specific foods groups, 59 (70.2%) participants wanted to eat more meat (chicken, mutton, fish, beef), 18 (21.4%) wanted to eat more dairy products, 61 (73.5%) wanted to have more cereals, 42 (50.0%) wanted to have more eggs, 53 (63.1%) and 61 (72.6%) desired to eat more vegetables and fruits respectively, 29 (34.5%) wanted to eat more pulses, while 26 (31.0%) and 40 (47.6%) wanted to have more junk food and sugary/sweet food items as depicted in Table 2.

### 3.6 Food security

The participants were asked if they had enough food at home to eat each day, where 34 (40.5%) and 38 (45.2%) reported sometimes and always respectively, while 9 (10.7%), 1 (1.2%) and 2 (2.4%) reported sometimes, rarely and never, respectively. Majority of the participants, 80 (95.2%) reported that all the household members had same quantity/quality of food, while 4 (4.8%) reported that males had more quantity of food as compared to females, where majority of such patients 3 out of 4 belonged to the intervention group as given in Table 2.

### 3.7 Malnutrition and related symptoms

As shown in Table 3, there were 28 (33.3%) participants who were extremely satisfied with their current health, 32 (38.1%) were moderately satisfied, 18 (21.4%) were somewhat satisfied, 5 (6.0%) were slightly satisfied while 1 (1.2%) was not at all satisfied with current health. There were only 6 (7.1%) participants who reported to get constipated sometimes, and only 15 (17.9%) participants reported to have small/dry stools rarely.

**Table 3:** Study participants’ responses to malnutrition-related symptoms.

Malnutrition-related symptoms	Overall (*n* = 84)
How satisfied are you with your current health?	
Not at all Satisfied	1 (1.2%)
Slightly Satisfied	5 (6.0%)
Somewhat Satisfied	18 (21.4%)
Moderately Satisfied	32 (38.1%)
Extremely Satisfied	28 (33.3%)
Do you experience Cramps in your legs?	
Never	19 (22.6%)
Rarely	5 (6.0%)
Sometimes	41 (48.8%)
Most of time	18 (21.4%)
Always	1 (1.2%)
Do you get breathless while walking/working/climbing stairs?	
Never	33 (39.3%)
Rarely	9 (10.7%)
Sometimes	36 (42.9%)
Most of time	6 (7.1%)
Always	0 (0%)
Do you get constipated?	
Never	68 (81.0%)
Rarely	10 (11.9%)
Sometimes	6 (7.1%)
Do you have small/dry stools?	
Never	68 (81.0%)
Rarely	15 (17.9%)
Sometimes	1 (1.2%)
Do you pass worms in your stools?	
Never	71 (84.5%)
Rarely	6 (7.1%)
Sometimes	7 (8.3%)
Have you noticed any recent changes to your skin, eyes or mouth?	
Yes	59 (70.2%)
No	25 (29.8%)
If yes, what type of changes?	
Nail Discoloration	1 (1.4%)
Dry Hair	5 (7.1%)
Hair loss	45 (63.4%)
Early Graying of hair	9 (12.9%)
Skin Itching	20 (28.6%)
Eye Dryness	4 (5.7%)
Mouth Ulcers	12 (17.1%)
Acne	13 (18.6%)

### 3.8 Associative relationship between BMI category and other socio-demographic variables

The chi-square test of association was used to determine the association between the 2 BMI categories; underweight and normal, and various other socio-demographic variables measured in the study, as shown in Table 4.

**Table 4:** Associative relationship between BMI categories and socio-demographic variables.

Socio-demographic variables	BMI categories		*p*-value
	Underweight (*n* = 73)	Normal BMI (*n* = 11)	
Family size (Mean ± SD)	7.7 ± 2.6	7.1 ± 1.8	0.432
Number of siblings (Mean ± SD)	4.3 ± 1.9	3.9 ± 1.5	0.486
Father’s monthly income (Mean ± SD)	20,305 ± 8,491	26,400 ± 7,893	0.127
Father’s occupation type			0.520
Private employee	45 (61.6%)	5 (45.5%)	
Self employed	18 (24.7%)	5 (45.5%)	
Government employee	8 (11.0%)	1 (9.1%)	
Unemployed	2 (2.7%)	0 (0.0%)	
House ownership			0.592
Rented	9 (12.3%)	2 (18.2%)	
Owned	64 (87.7%)	9 (81.8%)	
Housing structure			0.590
Kacha	8 (11.0%)	1 (9.1%)	
Pacca	59 (80.8%)	10 (90.9%)	
Katcha Pacca	6 (8.2%)	0 (0.0%)	
Water supply			
Piped water	65 (89.0%)	10 (90.9%)	0.971
Natural spring	1 (1.4%)	0 (0.0%)	
Well water	5 (6.8%)	1 (9.0%)	
Common water tank	1 (1.4%)	0 (0.0%)	
Canal/river	1 (1.4%)	0 (0.0%)	
Where do you go to defecate?			0.125
Household latrine	50 (68.5%)	10 (90.9%)	
Other latrine	23 (31.5%)	1 (9.1%)	
Do you have birds/animals in the house?			0.945
Yes	34 (46.6%)	5 (45.5%)	
No	39 (53.4%)	6 (54.5%)	
Do you have television at home?			0.772
Yes	56 (76.7%)	8 (72.7%)	
No	17 (23.3%)	3 (27.3%)	
Do you have any transport vehicle at home?			0.366
No	11 (15.7%)	0 (0.0%)	
Yes, Motorcycle	49 (70.0%)	9 (90.0%)	
Yes, Car	4 (5.7%)	1 (10.0%)	
Yes. Cycle	6 (8.6%)	0 (0.0%)	

Father’s education			0.529
Not able to read or write	23 (31.5%)	2 (18.2%)	
Less than primary	28 (38.4%)	6 (54.5%)	
Primary	16 (21.9%)	3 (27.3%)	
Secondary	6 (8.2%)	0 (0.0)	
Mother’s education			0.949
Not able to read or write	43 (58.9%)	6 (54.5%)	
Less than primary	23 (31.5%)	4 (36.4%)	
Primary	7 (9.6%)	1 (9.1%)	

All variables were not found to have a statistically significant association with BMI of the respondents. The source of water supply was found to be insignificantly associated with BMI (*p* = 0.971), as were father’s education level (*p* = 0.529), defecation place (*p* = 0.125), and family size (*p* = 0.432).

### 3.9 Associative relationship between BMI category and dietary patterns, preferences and food security

A higher number of participants belonging to underweight BMI category reported to have bad appetite as compared to participants with normal weight, such participants reported to consume 2 or less number of meals per day, some of them reported not taking snacks between meals, and a higher number of underweight participants reported that they skip meals most of the times as given in Table 5, but these differences were not statistically significant. A significantly higher number of participants belonging to the underweight BMI category were found to consume non-food items such as mud/clay as compared to those with normal weight (*p* = 0.046) as shown in Table 5.

**Table 5:** Association of BMI categories with dietary patterns, preferences and food security among study participants (*n* = 84).

	BMI categories		*p*-value
	Underweight (*n* = 73)	Normal BMI (*n* = 11)	
Dietary patterns			
Do you think that you have a good appetite?			0.351
Yes	61 (83.6%)	11 (100.0%)	
No	12 (16.4%)	0 (0.0%)	
How many meals do you have daily?			0.205
1 meal	3 (4.1%)	0 (0.0%)	
2 meals	63 (86.3%)	8 (72.7%)	
3 meals	7 (9.6%)	3 (27.3%)	
Do you take snacks between regular meals?			0.672
Never	2 (2.7%)	0 (0.0%)	
Rarely	6 (8.2%)	0 (0.0%)	
Sometimes	47 (64.4%)	7 (63.6%)	
Most of the time	16 (21.9%)	4 (36.4%)	
Always	2 (2.7%)	0 (0.0%)	
Do you skip meals?			0.460
Never	9 (12.3%)	3 (27.3%)	
Rarely	2 (2.7%)	0 (0.0%)	
Sometimes	57 (78.1%)	8 (72.7%)	
Most of time	5 (6.8%)	0 (0.0%)	
Which meal do you normally skip?			0.307
Breakfast	6 (9.1%)	0 (0.0%)	
Lunch	24 (36.4%)	5 (62.5%)	
Dinner	36 (54.5%)	3 (37.5%)	
How frequently do you skip your meals?			0.378
Never	7 (9.6%)	3 (27.3%)	
Rarely	5 (6.8%)	0 (0.0%)	
Sometimes	44 (60.3)	7 (63.6%)	
Most of time	16 (21.9%)	1 (9.1%)	
Always	1 (1.4%)	0 (0.0%)	
Do you eat junk/street food			0.623
Never	5 (6.8%)	0 (0.0%)	
Rarely	6 (8.2%)	2 (18.2%)	
Sometimes	35 (47.9%)	5 (45.5%)	
Most of time	27 (37.0%)	4 (36.4%)	
Food cravings			
Have you ever experienced cravings for any non-food items? e.g., mud, chalk, paper, coal etc.			0.470
Yes	3 (4.1%)	1 (9.1%)	
No	70 (95.9%)	10 (90.9%)	
If yes, what is the craving for?			0.046*
Mud/clay	3 (100%)	0 (0.0%)	
Chalk	0 (0.0%)	1 (100%)	
Food security			
Do you have enough food at home to eat each day?			
Never	2 (2.7%)	0 (0.0%)	0.216
Rarely	1 (1.4%)	0 (0.0%)	
Sometimes	8 (11.0%)	1 (9.1%)	
Most of time	36 (49.3%)	2 (18.2%)	
Always	26 (35.6%)	8 (72.7%)	
Have you ever had any food shortage in the house in the last year?			0.424
Never	46 (63.0%)	9 (81.8%)	
Rarely	14 (19.2%)	0 (0.0%)	
Sometimes	12 (16.4%)	2 (18.2%)	
Most of time	1 (1.4%)	0 (0.0%)	
Do all household members have the same number of meals?			0.10
Yes	71 (97.3%)	11 (100.0%)	
No, Males have more	2 (2.7%)	0 (0.0%)	
Do all Household members eat together at mealtimes?			0.563
Yes	66 (90.4%)	11 (100.0%)	
No, Females eat first	1 (1.4%)	0 (0.0%)	
No, Males eat first	6 (8.2%)	0 (0.0%)	
Do all the household members have the same quantity of food?			1.000
Yes	69 (94.5%)	11 (100.0%)	
No, Males have more	4 (5.5%)	0 (0.0%)	

*Means that the values were statistically significant. *p*-values of <0.05 were considered to be statistically significant.

### 3.10 Associative relationship between BMI category and malnutrition-related symptoms

It was also more number of participants belonging to underweight category reported to be slightly or not at all satisfied with their current health status, higher number of such participants to experience mostly or always experience cramps in legs, higher number of such participants reported to get breathless while walking/working/climbing stairs, there were more participants belonging to underweight group reporting that they notice worms in stools, and have recently notices changes in skin, eyes or mouth as compared to those with normal BMI as given in Table 6, but the differences were not statistically significant.

**Table 6:** Association of BMI categories with malnutrition-related symptoms (*n* = 84).

Variables	BMI categories		*p*-value
	Underweight (*n* = 73)	Normal BMI (*n* = 11)	
How satisfied are you with your current health?			0.637
Not at all Satisfied	1 (1.4%)	0 (0.0%)	
Slightly Satisfied	5 (6.8%)	0 (0.0%)	
Somewhat Satisfied	17 (23.3%)	1 (9.1%)	
Moderately Satisfied	27 (37.0%)	5 (45.5%)	
Extremely Satisfied	23 (31.5%)	5 (45.5%)	
Do you experience Cramps in your legs?			0.157
Never	14 (19.2%)	5 (45.5%)	
Rarely	5 (6.8%)	0 (0.0%)	
Sometimes	35 (47.9%)	6 (54.5%)	
Most of time	18 (24.7%)	0 (0.0%)	
Always	1 (1.4%)	0 (0.0%)	
Do you get breathless while walking/working/climbing stairs?			0.613
Never	27 (37.0%)	6 (54.5%)	
Rarely	8 (11.0%)	1 (9.1%)	
Sometimes	32 (43.8%)	4 (36.4%)	
Most of time	6 (8.2%)	0 (0.0%)	
Do you get constipated?			0.421
Never	58 (79.5%)	10 (90.9%)	
Rarely	10 (13.7%)	0 (0.0%)	
Sometimes	5 (6.8%)	1 (9.1%)	
Do you have small/dry stools?			0.654
Never	58 (79.5%)	10 (90.9%)	
Rarely	14 (19.2%)	1 (9.1%)	
Sometimes	1 (1.4%)	0 (0.0%)	
Do you pass worms in your stools?			0.553
Never	61 (83.6%)	10 (90.9%)	
Rarely	5 (6.8%)	1 (9.1%)	
Sometimes	7 (9.6%)	0 (0.0%)	
Have you noticed any recent changes to your skin, eyes or mouth?			0.846
Yes	51 (69.9%)	8 (72.7%)	
No	22 (30.1%)	3 (27.3%)	

### 3.11 Associative relationship between MUAC and other socio-demographic variables

There was no significant difference observed between MUAC and family size, number of siblings or father’s income.

MUAC was also found to have non-significant relationships with all of the socio-demographic variables and showed very weak correlation with these variables as well (Table 7).

**Table 7:** Association of MUAC with socioeconomic status.

	Overall (*n* = 84)	
	MUAC	
	Mean ± SD	*p*
Father’s occupation type		0.295
Private employee	19.6 ± 2.4	
Self employed	20.4 ± 2.4	
Government employee	19.8 ± 1.7	
Unemployed	17.3 ± 0.9	
House ownership		0.246
Rented	20.6 ± 2.4	
Owned	19.6 ± 2.4	
Housing structure		0.712
Kacha	19.2 ± 2.3	
Pacca	19.8 ± 2.5	
Katcha Pacca	20.1 ± 1.2	
Water supply		0.970
Piped water	19.8 ± 2.4	
Natural spring	20.6 ± 0.0	
Well water	19.4 ± 2.2	
Common water tank	18.5 ± 0.0	
Canal/river	20.0 ± 0.0	
Where do you go to defecate?		0.799
Household latrine	19.8 ± 2.4	
Other latrine	19.7 ± 2.3	
Do you have birds/animals in the house?		0.354
Yes	20.1 ± 2.6	
No	19.5 ± 2.2	
Do you have television at home?		0.657
Yes	19.8 ± 2.5	
No	19.5 ± 2.1	
Do you have any transport vehicle at home?		0.338
No	19.0 ± 1.7	
Yes, Motorcycle	20.1 ± 2.5	
Yes, Car	19.4 ± 3.2	
Yes. Cycle	18.7 ± 1.7	
Father’s education		0.674
Not able to read or write	19.9 ± 2.0	
Less than primary	19.7 ± 2.8	
Primary	20.1 ± 2.3	
Secondary	18.7 ± 2.0	
Mother’s education		0.541
Not able to read or write	19.7 ± 2.2	
Less than primary	20.1 ± 2.7	
Primary	19.1 ± 2.5	

### 3.12 Associative relationship between MUAC and dietary patterns, preferences and food security

Participants who reported to have bad appetite had slightly lower mean MUAC, participants taking one meal per day had low mean MUAC, participants who never or rarely took snacks in between meals had lower mean MUAC values, but these differences were not statistically significant as given in Table 8. Whereas significant difference was observed when mean MUAC was compared between participants reported to crave for mud or clay, where those who craved for mud/clay had significantly lower mean MUAC readings as compared to those experiencing no such cravings (*p* = 0.021). MUAC was also found to be statistically significantly associated with food shortage (*p* = 0.005).

**Table 8:** Association of MUAC with dietary patterns, preferences and food security among study participants (*n* = 84).

Variables	MUAC	
	Overall (*n* = 84)	
	Mean ± SD	*p*-value
Dietary patterns		
Do you think that you have a good appetite?		0.722
Yes	19.8 ± 2.5	
No	19.5 ± 1.2	
How many meals do you have daily?		0.287
1 meal	17.8 ± 2.8	
2 meals	19.8 ± 2.2	
3 meals	20.3 ± 3.2	
Do you take snacks between regular meals?		0.532
Never	18.5 ± 0.7	
Rarely	18.3 ± 2.9	
Sometimes	19.8 ± 2.4	
Most of the time	20.1 ± 2.2	
Always	19.7 ± 1.7	
Do you skip meals?		0.758
Never	20.4 ± 3.0	
Rarely	18.8 ± 1.6	
Sometimes	19.7 ± 2.3	
Most of time	20.1 ± 1.8	
Which meal do you normally skip?		0.290
Breakfast	19.3 ± 2.1	
Lunch	20.1 ± 2.6	
Dinner	19.3 ± 2.1	
How frequently do you skip your meals?		0.320
Never	20.9 ± 2.9	
Rarely	18.8 ± 2.1	
Sometimes	19.7 ± 2.4	
Most of time	19.3 ± 1.9	
Always	22.2 ± 0.0	
Do you eat junk/street food		
Never	19.1 ± 1.4	0.353
Rarely	20.1 ± 3.4	
Sometimes	19.3 ± 2.4	
Most of time	20.3 ± 2.2	
Food cravings		
Have you ever experienced cravings for any non-food items? e.g., mud, chalk, paper, coal etc.		
Yes	19.8 ± 2.2	0.971
No	19.8 ± 2.4	
If yes, what is the craving for?		0.021*
Mud/clay	18.7 ± 0.5	
Chalk	23.1 ± 0.0	
Food security		
Do you have enough food at home to eat each day?		0.069
Never	19.5 ± 2.5	
Rarely	21.0 ± 0.0	
Sometimes	19.8 ± 2.3	
Most of time	19.0 ± 2.2	
Always	20.6 ± 2.5	
Have you ever had any food shortage in the house in last one year?		0.005*
Never	20.2 ± 2.3	
Rarely	17.7 ± 1.6	
Sometimes	20.4 ± 2.5	
Most of time	20.0 ± 0.0	
Do all household members have the same number of meals?		
Yes	19.8 ± 2.4	0.124
No, Females have more	–	
No, Males have more	17.2 ± 2.5	
Do all Household members eat together at mealtimes?		0.866
Yes	19.8 ± 2.5	
No, Females eat first	18.5 ± 0.0	
No, Males eat first	19.8 ± 8.3	
Do all the household members have the same quantity of food?		0.331
Yes	19.8 ± 2.4	
No, Females have more	–	
No, Males have more	18.6 ± 0.4	

*Means that the values were statistically significant. *p*-values of <0.05 were considered to be statistically significant.

### 3.13 Associative relationship between MUAC and malnutrition-related symptoms

Participants who reported having experienced leg cramps most of the time had comparatively lower mean MUAC as compared to others, participants who noticed worms in their stool also had lower mean MUAC as compared to others, and participants who had noticed recent changes in their skin, eyes and mouth also had lower mean MUAC values, as given in Table 9. MUAC was significantly associated with leg cramping (*p* = 0.031) and with the occurrence of constipation evidenced by small/dry stools (*p* = 0.021).

**Table 9:** Association of MUAC with malnutrition-related symptoms.

Variables	Overall (*n* = 84)	
	Mean ± SD	*p*-value
How satisfied are you with your current health?		0.522
Not at all Satisfied	21.0 ± 0.0	
Slightly Satisfied	20.1 ± 1.7	
Somewhat Satisfied	19.1 ± 2.0	
Moderately Satisfied	19.7 ± 2.5	
Extremely Satisfied	20.3 ± 2.6	
Do you experience Cramps in your legs?		0.031*
Never	20.7 ± 2.7	
Rarely	18.8 ± 0.8	
Sometimes	20.0 ± 2.3	
Most of time	18.4 ± 2.1	
Always	21.0 ± 0.0	
Do you get breathless while walking/ working/ climbing stairs?		0.165
Never	20.5 ± 2.5	
Rarely	18.9 ± 2.3	
Sometimes	19.3 ± 2.3	
Most of time	19.5 ± 2.4	
Always	–	
Do you get constipated?		0.090
Never	19.9 ± 2.5	
Rarely	18.3 ± 2.0	
Sometimes	20.6 ± 0.9	
Do you have small/dry stools?		0.021*
Never	20.1 ± 2.4	
Rarely	18.2 ± 2.0	
Sometimes	21.0 ± 0.0	
Do you pass worms in your stools?		0.468
Never	19.9 ± 2.4	
Rarely	19.3 ± 2.3	
Sometimes	18.8 ± 2.1	
Have you noticed any recent changes to your skin, eyes or mouth?		0.293
Yes	19.6 ± 2.6	
No	20.2 ± 1.8	

*Means that the values were statistically significant. *p*-values of <0.05 were considered to be statistically significant.

## 4 Discussion

This study aimed to assess the various contextual and socio-demographic factors associated with the health status, particularly BMI levels and MUAC scores of school children. The study reported a prevalence of 86.9% students in the underweight category and mean BMI for age z-score value of −1.18 ± 1.05. Another study conducted in Punjab, Pakistan that assessed the BMI values of school children aged 10 years and above also found lower BMI values in the study participants as compared to the CDC and WHO growth chart references. The mean BMI value for 12 year olds in this study, which is also the mean age in our study, was 17.36 which falls in the underweight category (20). Another study conducted in Multan on school children also found anthropometric measurements consistent to our study. This study reported mean height, weight and BMI values to be 136.49 cm, 35.76 kgs and 18.40 kg/m^2^, respectively, as compared to our study’s values of 143.9 cm, 34.4 kg and 17.36 kg/m^2^. Both weight and height in both studies were also lower than the standard WHO references, mainly due to the pre-pubertal growth spurt in female children during this age (21). Pakistani children are at a higher risk of being underweight as compared to being overweight and obese and the prevalence of underweight children has steadily increased over the years. Another study found the prevalence to be 21.9% as compared to only 2.1% of school children falling below normal BMI values in China (22). The contributing factors include social, economic, education and environmental differences among countries, some of which have been assessed in our study.

One of these primary contextual factors is household income. Our study found the mean household income to be 20,781 rupees which is lower than the average Pakistani household income of 41,545 rupees (23). Another study conducted in the Philippines found significant associations between household income and weight for age z-scores in school children. Household income has direct effects on food security and availability of food to a family. Additionally, the quality and accessibility of foods is also compromised, which leads to negative nutritional impacts as depicted by lower weight for age z-score (WAZ) and height for age z-scores (HAZ), as well as lower BMI levels (24). Another important determinant of children’s nutritional status is the presence of latrines/washrooms. A Nigerian study reported that 6.3% of household had no proper washrooms and the most commonly available type of latrines were ‘pit latrines’ present in 93.7% households (25). A Bangladeshi study found open defecation practices in 12.4% of the study population and was significantly associated with poor hand washing practices leading to under nutrition in children (26). Our study reported the absence of proper latrines in 28.6% homes, which is higher than both the studies mentioned and has serious health and nutrition consequences for the study population.

Drinking water sources also poses serious health risks to the population and contaminated water results in 2.3 billion cases of water-borne diseases annually, with the majority being children. Water-borne disease prevalence in Pakistan is reported to be 20–40 and 70% of the water wells and springs, which are the main water source for the Pakistani population, are contaminated with industrial and bacteriological agents. This has resulted in increasing prevalence of diarrhea, dysentery and hepatitis, particularly in children. A study in district Bajaur, Pakistan reported tube wells, wells and hand wells to have the highest water quality while springs had the poorest water quality and had slightly acidic pH (27). In our study 89.3% of the population got drinking water from piped sources while only 1.2% reported spring water use, which depicts the use of comparatively safe drinking water. Our study also assessed parents’ literacy level and found 50% of fathers have primary level education and 41.6% mothers possess basic reading and writing skills. An Indian study assessed the relationship between parent’s education levels and children’s nutritional status and found undernourishment prevalence to be higher among children of uneducated parents, however the association was found insignificant (*p* < 0.11). However, parental education directly impacts a household’s income, living standard, food security and nutrition knowledge, it indirectly leads to poor nutritional status and development of children (28).

BMI was found to not be significantly associated with any of the socio-demographic and malnutrition variables. There was also no significant relationship between BMI and water supply source of the household (*p* = 0.971). These findings are again consistent with another study which found no significant association between water source and z-scores of children consuming water from wells, surface water and basic piped water supply. This suggests that merely improving a community’s water supply alone does not make significant impacts on improving children’s nutritional status (29).

However, when associated with dietary patterns and food preferences, BMI categories were found to be significantly associated with consumption of non-food items like mud and clay (*p* = 0.046). This recurrent consumption of non-nutritive substances is termed as ‘pica,’ which is an understudied feeding disorder among children. It has been linked with the development of eating disorders and subsequent malnutrition among children. Therefore, studies have focused on assessing the association between pica and BMI levels, and one such longitudinal study associated BMI z-scores with pica occurrence but found no significant association between the two, however, the study found a significant association between pica and eating difficulties which indirectly influence BMI levels in children (30).

Our study also assessed the association between MUAC and various socio-demographic variables but did not find significant relationships between them. We found family income and MUAC to have a statistically insignificant association between the two (*p* = 0.283). However, another Pakistani study found a significant relationship between family income and MUAC scores of children (*p* = 0.04), with a greater prevalence of lower MUAC scores in low-income families. This is also consistent with another south Indian study that found two thirds of children with low MUAC scores to have a lower household income (31). Our study also found no significant association between water supply and MUAC (*p* = 0.970). this finding is consistent with a Bangladeshi study that also found no significant associations between the source of drinking water and under nutrition evidenced by MUAC scores (*p* = 0.649). However, the same study did find a statistically significant relationship between water source for washing utensils and MUAC (*p* = 0.00) (32). Types of latrines such as household latrines or open defecation were also not found to have any association with MUAC levels (*p* = 0.799) as per our study’s findings. This is like a West African study which also found that the place of defecation had no effect on MUAC scores (*p* = 0.12). However, the study reported that social desirability bias when answering whether in-house of open latrines were used, may have resulted in a non-significant finding in this case (33). Father and mothers’ education levels were both found to be insignificantly associated with MUAC levels with *p*-values of 0.674 and 0.541, respectively. The same results were found in a Kenyan study which also reported mother’s education level to have no impact on child under nutrition (*p* = 0.60), although the prevalence of stunting was higher in children (31.3%) with mothers who had lower education levels (34).

When MUAC was associated with dietary patterns, food preferences and food security, significant relationships were found with consumption of mud/clay (*p* = 0.02) and with food shortage at home (*p* = 0.005). Another study conducted in the USA also found a positive association between food insecurity and low MUAC levels in children and found food insecurity during early childhood to influence eating patterns during this period of rapid growth and increased dietary requirement. Lower MUAC levels can be indicative of both acute and chronic undernutrition but in case of long term food insecurity, it is usually the latter, as in case of acute malnutrition, MUAC levels turn to normal within a year after food security is established (35). An Indian cross-sectional study in the resource poor settings also indicated that individuals with household food insecurity are likely to have inconsistent dietary patterns consequently increasing the likelihood of being underweight (31).

Any substantial relationship between undernutrition, demographic and socioeconomic variables could not be established except for a few indicators. The reason could be inadequate nutrition and health related education. A study on integrating nutrition-related education into the schools in low middle income countries suggests how undernutrition and overnutrition be addressed through essential nutrition-related interventions in the school environment by engaging the individuals, families and community members with multi-sectorial collaboration (32).

### 4.1 Study limitations and generalizability

Some of the study’s limitations included that the study was conducted in only two union councils of one subdivision, Gadap, of Gadap town in District Malir. The results of the study cannot be generalized as it was conducted in two schools only due to time and resource constraints, there was a limited study sample. Larger scale study might have led to stronger associations and correlations. Data collected were based on self-reports, which are subject to bias, since we had no means of verification beyond the girls’ own statements. Additionally, there were some concerns regarding safety and security.

## 5 Conclusion

Our findings show that majority of adolescent girls were underweight and were low height for age. MUAC and BMI were found to be associated with dietary patterns, food preferences and food insecurity. The high burden of underweight among school-going adolescent girls needs targeted approaches like nutrition education interventions to enhance awareness about healthy dietary behaviors, food preferences and ensuring access to safe, sufficient, and healthy diet.

Delivering essential nutrition-related interventions in the school settings should be considered to improve health and nutritional indicators. Nutrition and health related education play a crucial role in shaping adolescents’ capability to recognize their nutritional requirements, make healthier food choices, develop sound dietary habits, and manage undernutrition effectively.

A comprehensive approach is required, engaging not only the individuals but also involving families, community members, the educational system, health system, and other relevant sectors. By adopting a multifaceted strategy, we can address the various factors influencing adolescents’ nutritional and health outcomes.

## Data Availability

The raw data supporting the conclusions of this article will be made available by the authors, without undue reservation.
